# Assessing the Average Sodium Content of Prepacked Foods with Nutrition Declarations: The Importance of Sales Data

**DOI:** 10.3390/nu6093501

**Published:** 2014-09-04

**Authors:** Živa Korošec, Igor Pravst

**Affiliations:** Nutrition Institute, Tržaška cesta 40, Ljubljana 1000, Slovenia; E-Mail: ziva.korosec@nutris.org

**Keywords:** food composition, sodium, salt, processed foods, food labelling, nutrition claims, health claims, food supply

## Abstract

Processed foods are recognized as a major contributor to high dietary sodium intake, associated with increased risk of cardiovascular disease. Different public health actions are being introduced to reduce sodium content in processed foods and sodium intake in general. A gradual reduction of sodium content in processed foods was proposed in Slovenia, but monitoring sodium content in the food supply is essential to evaluate the progress. Our primary objective was to test a new approach for assessing the sales-weighted average sodium content of prepacked foods on the market. We show that a combination of 12-month food sales data provided by food retailers covering the majority of the national market and a comprehensive food composition database compiled using food labelling data represent a robust and cost-effective approach to assessing the sales-weighted average sodium content of prepacked foods. Food categories with the highest sodium content were processed meats (particularly dry cured meat), ready meals (especially frozen pizza) and cheese. The reported results show that in most investigated food categories, market leaders in the Slovenian market have lower sodium contents than the category average. The proposed method represents an excellent tool for monitoring sodium content in the food supply.

## 1. Introduction

A high dietary sodium intake is closely related to elevated blood pressure (BP), which is a major risk factor for the development of cardiovascular disease (CVD). Elevated BP causes almost 13% of all deaths worldwide, and in 2008, the global overall prevalence of elevated blood pressure in adults was around 40% [1]. However, the majority of CVD deaths attributable to BP occur at levels where drug therapy is not indicated [2,3]. Achieving a small downward shift in the distribution of BP in the whole population would achieve a surprisingly large CVD reduction [4,5].

Extensive evidence shows a consistently direct relationship between BP and salt/sodium intake [6,7]. In line with this evidence, several countries and health organizations have developed sodium-reduction recommendations. They have also provided evidence-based appraisals of how this might be achieved in specific settings [8,9,10,11,12,13]. The current targets of daily salt intake set by the World Health Organization (WHO) are 5 g/day or less [8]. Currently, daily salt intake in most countries varies between 9 and 12 g [14], which is well above WHO’s recommendations [15] and represents a major public health concern [16]. It was reported that a 4.6 g reduction in daily salt intake decreases BP by about 5.0/2.7 mmHg in hypertensive individuals and by 2.0/1.0 mmHg in normotensive people [7] and that a 5 g higher salt intake is associated with a 17% greater risk of total CVD and a 23% greater risk of stroke [3,17].

A reduction in population sodium intake may be achieved through a variety of approaches, *i*.*e*., health promotion and awareness campaigns, voluntary collaboration with food producers, the use of salt substitutes in households and in food manufacturing and also by regulatory means, including regulations on the use of nutrition and health claims. Barriers to progress in reducing sodium intake in the general population were investigated in a recent international study [18] revealing that, while sodium reduction is seen as important, over one-third of participants were not interested in salt/sodium reduction and the majority were unaware of the recommendations. Key strategies to reduce sodium intake should therefore focus on both lowering the availability of sodium in foods and raising consumer awareness about the importance of cutting sodium intake. However, it is very challenging to reduce sodium in food due to sodium’s specific functionality, including in terms of the flavor and the associated palatability of foods (*i*.*e*., the increase of saltiness, reduction of bitterness, enhancement of sweetness and other congruent flavors) [19]. A gradual reduction of sodium while maintaining palatability is therefore one of the best strategies. For example, it has been shown that such an approach enables at least a one-quarter reduction of the sodium content of bread, while maintaining consumer acceptance [20]. In the European Union (EU), the use of nutrition and health claims has been harmonized since 2006 [21,22], enabling the promotion of food with a variety of statements on food labelling and advertising, including a series of sodium-related nutrition and health claims [21].

To assess the efficiency of the above-mentioned approaches, robust and reliable evaluation methods are needed. While average sodium excretion in 24 h urine is recognized as the gold standard marker for measuring sodium intake in the population [23], this method cannot identify food sources of sodium. Therefore, different methods are used to assess population exposure and sodium availability in foods on the market.

Many processed foods have a relatively high sodium content and are recognized as the main contributors to population dietary sodium intake in both adults and children [24,25]. The sodium content of processed foods varies among food categories. The biggest food sources of sodium, besides plain salt used as a condiment, include processed meat, bread and bakery products, cheese and ready prepared meals [26,27,28]. Sodium intake reduction can be achieved by gradually reducing the sodium content of certain foods, bringing many beneficial effects for public health [29]. Past years have seen several successful initiatives and public health campaigns to reduce the sodium content of processed foods, but still more can be done in this area [14,30,31,32]. The consistent monitoring of sodium levels in processed foods over time is critical for assessing the voluntary collaborations with the food industry and the need for further public health activities.

In Australia, Webster *et al*. monitored the average sodium content in categories of processed foods known to significantly contribute to dietary salt intake and compared them to maximum target levels [26]. A comprehensive brand-specific food composition database was established to help monitor the sodium content of processed foods. The mean average sodium content of foods available within specific food categories was established, without weighting by sales data. A similar approach was taken in Slovenia by Hlastan Ribič *et al*., where sodium levels in specific key food categories (meat products, bread and bakery products) were determined using labelling information of prepacked foods, information from food composition databases and laboratory analyses of products available in households [33]. An innovative approach was introduced in the U.K. by Mhurchu *et al*., who used a combination of 12 months’ commercial consumer panel food-purchasing data with nutrient data for a more precise assessment of the sodium content of processed foods and for estimating the population’s exposure to sodium [27]. Their method is an example of academic-commercial collaboration [34] to protect and promote public health and is now being used to monitor compliance with the voluntary sodium targets for sodium content in the U.K. [35].

The objective of our study was to test a new approach to assessing the sales-weighted average sodium content in prepacked foods and to compare weighted and non-weighted average sodium contents in foods on the market. While a combination of consumer panel food-purchasing data with nutrient data enables the importance of particular food products to be weighted when assessing the average sodium content per food category, in many countries, consumer panels are currently not organized in a way that would enable the cost-effective use of such a methodology. We therefore tested an alternative method, whereby we used 12-month sales data provided by major national food retailers. Focusing on cost efficiency, our aim was to propose a new method to enable the efficient and regular monitoring of the food supply. Our second objective was to evaluate the use of sodium-related nutrition and health claims on products in those food categories recognized as major contributors to dietary sodium intake.

## 2. Experimental Section

### 2.1. Collection of Data

A point of departure was a representative national database on the availability of pre-packed foods on the market. The database was compiled using food labelling information from 6348 food products [36]. The food labelling information that was collected to create the database included labelled data on the nutritional composition, as well as the use of nutrition and health claims on labels. The sample included all foods on hand in selected food categories available in selected grocery stores at the time of sampling. Sampling was done in 2011 in four grocery stores of the most important retailers with a combined market share of 66% (one megamarket, two supermarkets and one discounter). The database was composed in agreement with the retailers where the data were collected. The selection of food categories was made according to Lalor *et al*. [37], with the addition of processed seafood, ready products, vegetable oils and plant-based imitations of milk and yoghurt. The following food categories were included: milk; yoghurts and fermented milk drinks; butter and spreads; cheeses; other dairy products; whole eggs; frozen fruits and vegetables; frozen ready meals; breakfast cereals; breads and similar products; fine bakery wares (biscuits); pasta and rice; fruit juice and smoothies; soft drinks and water; teas; peas, beans and lentils; processed meats; processed seafood; ready meals, full meals; ready meals, other; vegetable fats and oils; plant-based imitations of milk and yoghurt. The sample of foods therefore does not include some food categories, *i*.*e*., food supplements, alcoholic drinks, confectionery, unprocessed cereals and snacks. For the purpose of this research, foods in the database were classified in further subcategories, including butter; vegetable fat spreads; milk-based spreads; vegetable-based spreads; mayonnaise; frozen pizza; frozen savory dishes; frozen sweet dishes; breads; toasts; crackers; wafers; dry cured meat; fresh salamis and hot dogs; and canned meats.

In the next stage, the retailers were asked to provide 12-month, country-wide sales data for each product included in the database (January, 2011–December, 2011). Ensuring proper data handling, we were able to obtain sales data from retailers covering over 62% of the national market. The sales data only referred to the national market and presented sales of food products for the same year in which the abovementioned food composition database was compiled. This was arranged on the condition that the results would not reveal sales data of any particular retailer. The final sample of foods used in this study is composed of 5104 pre-packed food products for which 12-month sales data were available (80% of the sample). The sales data were given in universal form, including the European/International Article Number (EAN barcode), description of the product, number of products sold per year and the quantity of food (kg/L) per packaging. The matching of foods between the databases was performed using EAN barcodes.

### 2.2. Calculation of the Average Sodium Content per (Sub)Category

Average sodium content in available prepacked foods (SCA; in mg of sodium per 100 g/mL) was calculated for selected food categories and subcategories. SCA values present the average sodium content in all products within a specific (sub)category for which sodium/salt levels were labelled.

Average sodium content in sold prepacked foods (SCS; mg per 100 g/mL) was also calculated for selected food categories and subcategories. Since a number of the same food products was available in different stores, the sales data provided by the grocery stores were combined to calculate the number of products sold per year for each food product in the database. Using data on the content of food per packaging, we calculated the amounts (kg/L) of products sold per year. In the next stage, the total content of sodium in products sold in a specific food category (kg) was calculated using labelled sodium/salt levels. Finally, SCS was calculated in mg of sodium per 100 g/mL to ensure comparability with SCA values. SCS values present the average sodium content in all products sold within a specific category for which sodium/salt levels were labelled.

### 2.3. Assessment of the Use of Sodium-Related Nutrition and Health Claims

The abovementioned database also included data on the use of nutrition and health claims on food labels. We analyzed the database to investigate the use of sodium-related nutrition and health claims in the sample of pre-packed food products for which 12-month sales data were available. We determined how common it is to use such claims and what kind of sodium-related claims are most commonly found on the products.

### 2.4. Statistical Analyses

Statistical analyses were performed using Microsoft Excel 2013 (Microsoft Corporation, Redmond, DC, USA). For average sodium content in available prepacked foods (SCA), the standard deviation (SD) was calculated. Average sodium content in sold prepacked foods (SCS) was given as exact values, and therefore, no SD is presented.

## 3. Results

**Figure 1:**
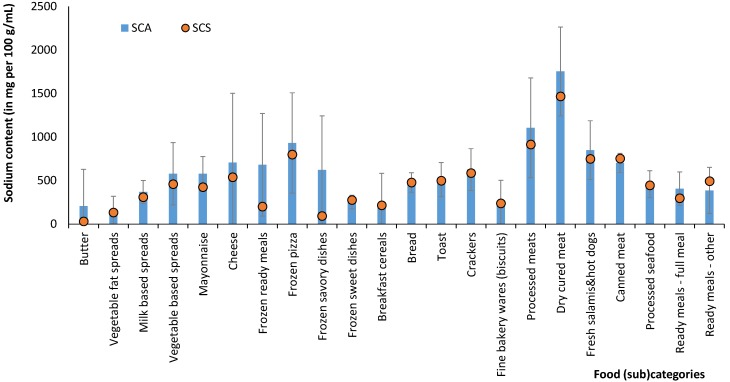
Comparison of average sodium content in available (SCA) and sold (SCS) prepacked foods for selected food (sub)categories.

Using a representative national database on the availability of pre-packed foods on the market and 12-month, country-wide sales data for each product included in the database (*n* = 5,104), we calculated average sodium content in available prepacked foods (SCA) and in sold prepacked foods (SCS) for selected food categories. The results are presented in Table 1 and Figure 1. Food categories with the highest sodium content were processed meats (particularly dry cured meat), ready meals (especially frozen pizza) and cheese. In some food categories, the standard deviation was more distinct than in others due to the very different composition of foods in the food category. The biggest differences between SCA and SCS values were observed in the categories of butter and frozen savory dishes (ready meals).

**Table 1:** Average sodium content in available (SCA) and sold (SCS) prepacked foods for selected food categories.

Food Category	*N*	% LSC ^1^	Average Sodium Content (mg per 100 g/mL)		SAR: SCS/SCA Ratio ^4^
			SCA ± SD ^2^	SCS ^3^	
Milk	53	36%	42 ± 4	41	98%
Yoghurt and fermented milk drinks	294	50%	47 ± 41	42	91%
Butter and spreads	196	47%	345 ± 309	194	56%
Butter	36	28%	205 ± 423	31	15%
Vegetable fat spreads	39	92%	158 ± 160	133	84%
Milk-based spreads	46	26%	368 ± 131	309	84%
Vegetable-based spreads	39	44%	578 ± 357	459	79%
Mayonnaise	36	47%	576 ± 200	424	74%
Cheese	335	11%	707 ± 795	539	76%
Other dairy products	138	26%	49 ± 24	46	94%
Frozen fruit and vegetables	111	32%	37 ± 79	46	127%
Frozen ready meals	239	17%	681 ± 590	202	30%
Frozen pizza	23	70%	932 ± 577	799	86%
Frozen savory dishes	155	11%	622 ± 622	93	15%
Frozen sweet dishes	61	11%	253 ± 77	276	109%
Breakfast cereals	276	82%	228 ± 355	215	94%
Breads and similar products	297	42%	510 ± 257	510	100%
Bread	121	25%	477 ± 112	478	100%
Toast	29	55%	511 ± 196	499	98%
Crackers	122	52%	626 ± 240	587	94%
Fine bakery wares (biscuits)	323	37%	250 ± 253	239	95%
Soft drinks	312	41%	20 ± 35	21	104%
Energy drinks	29	69%	46 ± 27	41	88%
Isotonic and sport drinks	20	90%	44 ± 22	26	59%
Processed meats	429	13%	1,106 ± 573	915	83%
Dry cured meat	107	16%	1,754 ± 510	1467	84%
Fresh salamis & hot dogs	201	17%	849 ± 339	749	88%
Canned meat	121	4%	702 ± 111	753	107%
Processed seafood	237	12%	457 ± 155	445	97%
Ready meals, full meal	96	51%	407 ± 192	297	73%
Ready meals, other	133	21%	386 ± 264	492	127%
Milk imitates	30	100%	39 ± 25	30	77%
Yoghurt imitates	32	88%	42 ± 28	39	94%

Notes: ^1^ % LSC, percentage of products with labelled sodium content; ^2^ SCA ± SD, average sodium content in available prepacked foods (SCA) ± the standard deviation (SD); ^3^ SCS, average sodium content in sold prepacked foods; ^4^ SAR, the ratio between SCA and SCS.

In the whole sample of 5104 foods, only 52 products (1%) were labelled with sodium-related nutrition claims, and no sodium-related health claims were found. The majority of sodium-related nutrition claims were found on breakfast cereals (*n* = 18) and soft drinks and water (*n* = 16). Only five products in bread and similar products and two products within processed meats were labelled with a sodium-related nutrition claim, while no such claims were found among cheeses.

## 4. Discussion

While in many countries sodium-reduction targets have been set for a large number of processed food categories, efficient assessment and monitoring are essential for evaluating related progress. Targeting sodium reduction in a small number of food categories and focusing on products sold in the highest volumes could lead to large decreases in sodium available for consumption and, therefore, to gains in public health [27]. A comparison of the average sodium content of specific food (sub)categories with (SCS) and without the use of weighting by sales (SCA) provides very useful information on the sodium content of the foods of market leaders in comparison to the average sodium content in a particular food category. In our study, we observed that in most food categories, SCS levels are lower than SCA levels, suggesting that market leaders in the Slovenian market have lower sodium contents than the category average (Table 1, Figure 1). One reason for such an observation could lie in consumers’ awareness of health risks related to high salt/sodium intake, but in general, this should be attributed to a combination of different factors, including price, brand, taste and texture expectations and consumers’ experiences with a particular food product. Our observations are only partially in line with the observations of Mhurchu *et al*., who performed a similar study in the U.K. market, but with a different methodological approach [27]. In a series of food groups, they observed that purchase-weighted means were higher than unweighted means; such food categories included bread (weighted *vs*. unweighted mean sodium content ratio (SAR, the ratio between SCA and SCS): 107%), processed meat (118%), breakfast cereals (126%) and milk (11%). On the other hand, below-average sodium levels were found in a series of other categories, e.g., in fish and fish products (92%) and processed vegetables (88%). Their study was based on 12-month consumption data for 21,108 British households collected through a commercial consumer panel. Panel members scanned and recorded all food and drink purchases brought into their homes. Data were available for over 40,000 food products. In contrast, our study is based on sales data provided by market leading food retailers, covering 62% of the national market (composed of over 800,000 households, [38]). The estimate of the percentage of the market covered by our study was made on the basis of available market share data of the retailers. We should mention significant differences in the market share of those retailers for different food categories. While in some food categories, sales of prepacked foods in retail stores represent the majority of sales, in other food categories, other sale routes are also significant. To evaluate this, we compared 12-month sales data of foods in our dataset with official national statistics on the average annual quantity of food and beverages consumed per household member [39]. The calculations revealed that our dataset covered the majority of purchased soft drinks (87%), about half of milk (41%), yoghurts (55%), butter (57%) and vegetable fat spreads (50%) and only 7% of bread, which is mostly sold in Slovenia as either a non-prepacked product or is widely available in specialized local bakery stores. Nevertheless, also in such food categories with a lower market share of prepacked products in retail stores, the volumes of products included in our calculations are significant. For example, our sample of bread exceeded the annual sales of 5000 tons of bread.

The highest sodium content was found in processed meat products (915 mg/100 g), particularly in dry cured meat (1467 mg/100 g). Our results are generally in line with the average sodium content of foods in Slovenia determined by Hlastan Ribič *et al*. using the Household Budget Survey and labelling information of prepacked foods and a combination of information from food composition databases and laboratory analyses of products available in households ([33], Table 2), except for dry cured meat, where we observed 18% lower average sodium levels. The observed SAR ratio in the whole category was 83%, quite lower than in the U.K. market (118%, [39]). Canned meat was a notable exception with an SAR ratio of 107% (98% in the U.K. study). We should also note the relatively low level of labelling of the sodium content of processed meats, particularly compared to certain other investigated food categories. Nutrition declarations are currently only compulsory in the EU for foods labelled with nutrition and/or health claims [21], and the proportion of foods with labelled nutrition information varies significantly between different food categories and countries. Interestingly, among all EU countries, the smallest proportion of foods with a nutrition declaration was found in Slovenia [40]. By the end of 2016, the inclusion of salt content on labelling will become mandatory for all processed prepacked foods in the EU [41]. This will enable the inclusion of a number of additional available foods to further studies and significantly contribute to the even greater reliability of the results.

**Table 2:** Comparison of average sodium content of foods determined using labelling information of sold prepacked foods (SCS) and a combination of information from food composition databases and laboratory analyses of products available in households.

Food Category	Average Sodium Content (mg per 100 g/mL)		Food Category (Hlastan Ribič *et al*. [33])
	SCS ^1^	SCLA ^2^	
Bread	478	455	Brown bread
		506	Mixed bread
		565	White bread
Cheese	539	694	Cheese
Dry cured meat	1467	1800	Dry meat
		2445	Prosciutto
Fresh salamis and hot dogs	749	837	Various sausages
		699	Frankfurters (hot dog)
		839	Various salamis

Notes: ^1^ SCS, average sodium content in sold prepacked foods for selected food (sub)categories; ^2^ SCLA, average sodium content determined in selected food (sub)categories using the Household Budget Survey and a combination of information from food composition databases and laboratory analyses of products available in households (Hlastan Ribič *et al*. [33]).

High sodium levels were also observed in ready meals, particularly in frozen pizza (SCS: 799 mg/100 g; SAR: 86%). Generally, we observed relatively low SAR ratios, e.g., 30% in the whole frozen ready meals category and 73% in full ready meals (Table 1). Considering usual portion sizes, ready meals represent a major source of dietary sodium for regular consumers of such foods. Our observations show that the sodium content of market leaders is below-average. On the contrary, an SAR ratio of 102% was observed for pizzas and ready meals in the U.K. study [39].

Cheese is also considered a significant source of dietary sodium. While the average observed sodium level (SCA) was 707 mg/100 g, the sales-weighted sodium level was considerably lower (SCS: 539 mg/100 g; SAR: 76%). This is 22% different to the previously reported average sodium content of cheese in the Slovenian market (694 mg/100 g, Table 2) [33] and 5% different compared to the U.K. market (568 mg/100 g, SAR: 122%) [39].

Over 80% of the Slovenian population consumes at least one portion of bread on a daily basis [42]. The average sodium level in bread was 478 mg/100 g (SCS). This is in line with previous reports (455, 506 and 565 mg/100 g for brown, mixed and white bread, respectively; Table 2). The observed SAR ratio was 100%. In the U.K. study, the weighted mean sodium level in bread was about 10% lower (426 mg/100 g; SAR: 107%) [39].

The most extreme differences between sales-weighted and unweighted sodium content levels were observed for butter and frozen savory dishes (SAR: 15%). An additional validity check confirmed that the data on the nutritional composition of foods in those food categories were correct. To gain a further insight into this phenomenon, we evaluated the importance of each food product in the database for the assessment of average sodium content per category. While salted butter is available in the Slovenian market, sales of it are insignificant in comparison with non-salted butter. The sales-weighted SCS values are therefore significantly lower (31 *vs*. 205 mg/100 g). Similarly, a high sales volume of specific products with low sodium content was noted in the category of savory frozen dishes, reducing the SAR ratio in this category.

Food labelling presents one of the strategies for improving consumers’ awareness about sodium content in foods; however, the possibilities for food producers to communicate the reduced sodium content of foods are very limited. Currently, five different sodium-related nutrition claims may be used in the European Union: (1) low sodium; (2) very low sodium; (3) sodium-free; (4) no added sodium; and a comparative claim, (5) reduced sodium; together with other statements with the same meaning for the consumer [21]. In addition, foods with a low or reduced content of sodium can be labelled with the health claim “reducing consumption of sodium contributes to the maintenance of normal blood pressure”.

The database of foods used in this study was actually compiled to support monitoring of the use of nutrition and health claims on the market. Having access to labelling information for all products in the database, we were able to evaluate the use of sodium-related nutrition and health claims and to assess whether differences between observed SCA and SCS values can be explained with the use of such claims. We particularly focused on food categories recognized as major contributors to dietary sodium intake. In the whole sample of 5,104 foods, only 52 products (1%) were labelled with sodium-related nutrition claims and no sodium-related health claims were found. Such a prevalence is quite lower than in Ireland (3.8%) [37] or in Canada (4.5%) [43]. The sodium-related claims most frequently observed were low sodium (on 18 products) and no added sodium (17 products). The majority of claims were found in the category of breakfast cereals (18 products). Use of sodium-related nutrition claims within food categories known to be major contributors to dietary sodium intake was very rare. For example, only five products in bread and similar products and two products within processed meats were labelled with a sodium-related nutrition claim, while no such claims were found among cheeses. Evaluation of the sodium content of all available products within those categories revealed that the majority of products does not comply with the strict criteria for using the nutrition claim low sodium (less than 0.12 g of sodium per 100 g/mL). In fact, no products within bread, processed meats and cheese were eligible for such a claim. Therefore, the only possibility for informing consumers about lowering the sodium content was to use a comparative claim on reduced sodium, where at least a 25% reduction of sodium should be assured. Only four products were labelled with such a claim. This indicates that, in most cases, food producers that are gradually reducing the sodium content of their foods are unable to communicate this to consumers.

A major strength of this study is its employment of academic-commercial collaboration, which enabled cost-effective use of sales data to assess average sodium contents in various food categories. Food retailers operate with detailed data on sales of any food products available, and the use of such data does not incur any additional costs. Such an approach facilitates the assessment of sodium availability in foods on the market in unprecedented dimensions, particularly if the partnership combines a series of different retailers accounting for the majority of market share, as was the case in this study. Compared to methods where food recalls or diaries are used, the use of 12-month sales data allows more precise estimates of the average sodium content in specific food categories and efficient monitoring of changes in the food supply. Since no additional efforts (other than purchasing) are needed from consumers, there is also no risk of under-reporting purchases of specific foods. Further, considering that the majority of the market is included, the habits of consumers of all demographic backgrounds are well represented.

A comprehensive database on the composition of foods available on the market is needed to perform such studies. To enable the food supply to be monitored over time, such a database should be regularly updated and available for use without restrictions [44]. An international collaborative project is underway within the Global Food Monitoring Group in which the nutritional composition of processed foods is surveyed using standardized methodology on a yearly basis in a number of countries [45,46], although it does not yet cover Slovenia. Such an approach enables efficient monitoring of the food supply to support governments, industry and communities to develop strategies to fight against food-related non-communicable diseases [47], particularly when connected with food sale/consumption data.

Given that food labelling data are mostly in line with the actual sodium content of the product, such data represent valid and easily accessible information for further estimations. A key limitation of our study is that in Slovenia, a significant proportion of prepacked foods is currently not labelled with a nutrition declaration. This phenomenon was previously reported [40], although the situation is changing, and the labelling of salt content will become mandatory on all prepacked processed foods by the end of 2016 [41]. This will enable the inclusion of several additional foods to further studies and significantly improve the quality of such studies in the future. When this has been resolved and complete databases of foods become available, our methodology will also enable the importance of particular food categories in dietary sodium intakes to be estimated. Other limitations of our study mostly relate to the food classification system, the exclusion of non-processed foods and processed foods purchased from local providers (*i*.*e*., local bakery stores) and by the fact that not all purchased food is actually consumed. This study was performed using a food database that had been compiled for a different purpose and, considering the pilot nature of the study, we did not carry out a re-categorization of all products in the database. Careful selection of a food categorization system in future studies should enable an easier comparison of results from different studies. Close collaboration and mutual trust between academia and food retailers is a precondition for using our methodology successfully, and this could be a significant limitation in some environments. In our case, the trust was gained through support for our efforts from the authorities, open discussions on issues related to data sharing and by our strict commitment to data protection. To prove this commitment to retailers, we were the first research organization in Slovenia to be accredited for its information security management system according to ISO 27001.

## 5. Conclusions

We have shown that a combination of 12-month food sales data provided by food retailers covering the majority of the national market and a comprehensive food composition database compiled using food labelling data represent a robust and cost-effective approach to assess the sales-weighted average sodium content of prepacked foods. Such an approach is a useful tool for monitoring the sodium content of processed foods on the market and shifts in purchasing behavior. Food categories with the highest sodium content were processed meats (particularly dry cured meat), ready meals (especially frozen pizza) and cheese. In some food categories, the standard deviation was more distinct than in others due to the different composition of specific products on the market. The biggest differences between the weighted and non-weighted average sodium content of prepacked foods were observed in the categories of butter and frozen savory dishes (ready meals). The reported results show that in most investigated food categories, market leaders in the Slovenian market have lower sodium contents than the category average. Analyses of the use of salt-related claims on foods on the market showed that only 1% of the investigated food products (whole sample) were labelled with sodium-related nutrition claims, and no sodium-related health claims were found. The sodium-related claims most frequently observed were low sodium and no added sodium, mainly found on products in the category of breakfast cereals.
